# Identification and Characterization of an Antibacterial Type VI Secretion System in the Carbapenem-Resistant Strain *Klebsiella pneumoniae* HS11286

**DOI:** 10.3389/fcimb.2017.00442

**Published:** 2017-10-12

**Authors:** Lu Liu, Meiping Ye, Xiaobin Li, Jun Li, Zixin Deng, Yu-Feng Yao, Hong-Yu Ou

**Affiliations:** ^1^State Key Laboratory of Microbial Metabolism, Joint International Laboratory on Metabolic & Developmental Sciences, School of Life Sciences and Biotechnology, Shanghai Jiao Tong University, Shanghai, China; ^2^Department of Respiratory Medicine, Shanghai Pulmonary Hospital, Tongji University School of Medicine, Shanghai, China; ^3^Laboratory of Bacterial Pathogenesis, Department of Medical Microbiology and Parasitology, Institutes of Medical Sciences, Shanghai Jiao Tong University School of Medicine, Shanghai, China

**Keywords:** type VI secretion system, *Klebsiella pneumoniae*, antibacterial, phospholipase, antibiotic stress

## Abstract

The type VI secretion system (T6SS) is a class of sophisticated cell contact-dependent apparatus with anti-eukaryotic or anti-bacterial function. *Klebsiella pneumoniae* is one of the most common bacterial pathogens with resistance to the carbapenem antibiotics. However, little is known about the antibacterial T6SS in *K. pneumoniae*. Using core-component protein searches, we identified a putative T6SS gene cluster on the chromosome of the carbapenemase-producing *K. pneumoniae* (CRKP) strain HS11286. Intraspecies and interspecies competition assays revealed an antibacterial function of the HS11286 T6SS. The phospholipase Tle1^KP^ was found to be an effector protein that is transferred by T6SS. The overexpression of this effector gene in the periplasm caused severe growth inhibition of *Escherichia coli*. A sub-inhibitory concentration of β-lactam antibiotics stimulated the expression and secretion of the HS11286 T6SS and enhanced T6SS-dependent killing. It suggested that the antibiotics might be an impact factor for the T6SS secretion and antibacterial activity.

## Introduction

The type VI secretion system (T6SS) is structurally related to the cell-puncturing device of tailed bacteriophages and functions as a contractile injection machinery that perforates eukaryotic and prokaryotic target membranes (Pukatzki et al., 2006; Kapitein and Mogk, 2013; Alteri and Mobley, 2016). T6SS gene cluster contains the core component genes and variable regions that encode effector and immunity proteins (Zhang et al., 2012; see the comprehensive review of Russell et al., 2014). The VipA and VipB proteins form a dynamic tubular sheath that switches between extended and contracted states within the bacterial cytosol (Basler and Mekalanos, 2012). Upon contraction of the cytoplasmic VipA/VipB tube, the hemolysin-coregulated protein (Hcp) capped with valine-glycine repeat protein G (VgrG) were ejected from the bacterium (Bönemann et al., 2009; Leiman et al., 2009; Basler and Mekalanos, 2012). ClpV (a class of ATPases) is required to disassemble the contracted VipA/VipB sheath (Bönemann et al., 2009; Basler and Mekalanos, 2012). Two core proteins, Hcp and VgrG, have frequently been used as markers to investigate the T6SS translocation of secreted effectors (Pukatzki et al., 2006, 2009; Li et al., 2016).

T6SS occur in many pathogenic bacteria and are implicated in virulence in important pathogens, including *Pseudomonas aeruginosa* (Mougous et al., 2006), *Vibrio cholerae* (Pukatzki et al., 2006), *Edwardsiella tarda* (Zheng and Leung, 2007), *Burkholderia mallei* (Schell et al., 2007), *Burkholderia cenocepacia* (Aubert et al., 2008), and *Aeromonas hydrophila* (Suarez et al., 2008). In several cases, the host-pathogen interaction of such “anti-eukaryotic” T6SSs resulted in disruption of the actin cytoskeleton (Pukatzki et al., 2007; Aubert et al., 2008; Suarez et al., 2010). Recently, T6SS has been reported as weaponry in interbacterial warfare (Hood et al., 2010; see the detailed review of Hood et al., 2017). It provides a fitness advantage by delivery of effector proteins to hydrolyze cell walls, cell membranes, and nucleic acids of opponent bacteria (Kapitein and Mogk, 2013; Jiang et al., 2014; Russell et al., 2014). Each of these effectors exhibited toxicity to bacteria and was located adjacent to the genes encoding proteins that conferred immunity to the toxin, thereby preventing self-intoxication (Russell et al., 2011; Zhang et al., 2012). Among these, membrane-targeting effectors were identified and found to have lipase activities (Dong et al., 2013; Russell et al., 2013). They are classified into five families, Tle1 to Tle5 (Russell et al., 2014).

The bacterial species *Klebsiella pneumoniae* is an increasingly important human pathogen. Dramatic increases in the levels of multidrug resistance associated with this species pose an emerging global problem (Broberg et al., 2014), particularly for carbapenemase-producing *K. pneumoniae* (CRKP; Tzouvelekis et al., 2012; Cubero et al., 2015). The *in silico* analysis showed that the putative T6SS gene clusters were present in the complete genome sequence of *K. pneumoniae* NTUH-K2044, MG78578, and Kp342 (Sarris et al., 2011). Furthermore, a T6SS in the hypervirulent *K. pneumoniae* strain Kp52.145 with the K2 capsular serotype transfers the effector phospholipase D1 (Tle5, PLD1), which has been reported as a novel virulence factor (Lery et al., 2014). However, little is known about the antibacterial function of T6SS in *K. pneumoniae* (Hood et al., 2017). A recent finding suggested that the rice pathogenic bacterium *Acidovorax avenae* strain RS-1 exposure to the β-lactam antibiotics enhanced the virulence of T6SS (Li et al., 2016). Thus, a better understanding of the role of T6SS in CRKP under antibiotic stress is warranted.

We have recently reported the complete genome of *K. pneumoniae* HS11286, an ST11, carbapenemase (KPC)-2-producing clinical isolate collected in 2011 from the sputum specimen of an inpatient in Shanghai, China (Liu et al., 2012). *K. pneumoniae* ST11 is a dominant KPC-producing clone in China and is closely related to the worldwide-dominant CRKP clone ST258. In this study, we annotated an entire T6SS gene cluster of *K. pneumoniae* HS11286. The anti-bacterial function of this T6SS was subsequently investigated with bacterial competition assays and overexpression of the effector gene *tle1*^*KP*^ in *E. coli*.

## Materials and methods

### Ethics and consent

In this study, sampling collection of patient sputum is a routine hospital procedure. As such, verbal informed consent was obtained from the volunteer. The bacterial sample and data sheet were anonymized. This study protocol, including the verbally informed consent procedure, was approved by the ethics committee of the School of Life Sciences & Biotechnology, Shanghai Jiao Tong University, China.

### Bacterial strains and growth conditions

The strains, plasmids, and primers used in this study are listed in Supplementary Tables 1–3, respectively. *K. pneumoniae* strain HS11286 was used as the sample strain in all experiments in this study unless otherwise noted in the figure legend. *E. coli* strain DH10B (streptomycin resistant) and *E. coli* strain BL21 were used for cloning and expressing the target gene, respectively. Unless stated otherwise, bacteria were grown in Luria-Bertani (LB) broth at 37°C with shaking motion (220 rpm). The antibiotic concentrations used were 100 μg/mL ampicillin, 25 μg/mL chloramphenicol, 100 μg/mL streptomycin, 50 μg/mL kanamycin, 200 μg/mL hygromycin, and 50 μg/mL apramycin. Isopropyl β-D-1-thiogalactopyranoside (IPTG) was added at a final concentration of 0.5 mM to induce the expression of the T7 promoter.

### Identification of T6SS loci in *K. pneumoniae* strain HS11286

The nucleotide sequence and annotation of the completely sequenced *K. pneumoniae* HS11286 chromosome were downloaded from the NCBI RefSeq Project (accession number NC_016845.1). The putative T6SS gene clusters were identified and aligned by using the web-based tool VRprofile (http://bioinfo-mml.sjtu.edu.cn/VRprofile/; Li et al., 2017). Briefly, it performs the Hidden Markov Model (HMM)-based detection of T6SS core components encoded by *K. pneumoniae* chromosome sequences. The annotated T6SS core components are searched against the VRprofile-specific HMM profiles. Significant hits within a defined gene distance are subsequently grouped to detect putative T6SS gene clusters. The minimum number of the colocalized T6SS core components is five to exclude false positives. Besides, VRprofile also employs BLAST searches to examine the rearranged architecture of a given T6SS gene cluster among multiple *K. pneumoniae* genomes under investigation. It aids identification of putative effectors and immunity protein genes inserted within or around the T6SS core components.

### Bacterial competition assays

Bacterial strains were grown overnight to stationary phase and suspended in LB medium. We washed strains with 10 mM MgSO_4_ and diluted to an optical density at OD_600_ of 0.2 before mixing at a ratio of 1:1 (attacker: prey). Cells of each strain were spotted on a sterile 0.22 μm filter (Millipore) on LB solid medium (casein tryptone 10 g/L; yeast extract 5 g/L; NaCl 5 g/L; agar 3%). Competitions were incubated for 5 h at 37 °C. Each group of bacteria was harvested, and serial dilutions were grown in selective culture medium containing 200 μg/mL hygromycin. Cells of each strain were grown overnight. The CFU per milliliter of the surviving prey strain was measured by counting single clones. The prey strain was the Δ*tle1*^*KP*^Δ*tli1*^*KP*^::hph of *K. pneumoniae* HS11286 carrying the hygromycin-resistance gene (Supplementary Table 1). The fatality rate was calculated as follows:

(1) $$Fatality\;rate=\frac{CFUcompetition}{CFUcontrol}\times 100\%$$

where *CFU*_*competition*_ is the difference between the CFU of the surviving prey strain (Δ*tle1*^*KP*^Δ*tli1*^*KP*^::hph) that was co-incubated with attacker strain Δ*vipA* and the CFU of the same prey strain when co-incubated with wild type attacker strain. The *CFU*_*control*_ is the CFU of the prey strain (Δ*tle1*^*KP*^Δ*tli1*^*KP*^::hph) without growth competition by any attacker strain.

### Mutagenesis

For gene mutation, the gene was first replaced by an FRT site-flanking *hph* cassette (Supplementary Figure 1) via lambda red recombination, resulting in an intermediate strain. Then, the *hph* cassette was eliminated via Flp-FRT recombination to obtain a markerless in-frame indel mutant strain (Supplementary Figure 2). In-frame deletion of a gene was performed as described previously (Hoang et al., 1998; Chaveroche et al., 2000; Bi et al., 2015).

### Western blot analysis

An Hcp antibody was produced by B&M Biotech (Beijing, China). Before immune injection into rabbits, we checked whether the rabbit serum gave an immune response to the whole protein extract of HS11286 at the target protein length to avoid a false-positive response. Rabbits were grown in specific-pathogen-free (SPF) conditions to ensure a non-specific immune response. Upon receiving the antibody, we first tested its specificity by blotting against Hcp protein. The specificity of antibody was shown in Supplementary Figure 3. For western blot, proteins were resolved on a precast 15% SDS/PAGE gel and transferred to a PVDF membrane (Millipore) by electrophoresis. The membrane was then blocked with 5% skimmed milk for 1 h at room temperature and incubated with primary antibodies (MBL Biotech) at 4°C overnight. The membrane was washed three times with TBST buffer (50 mM Tris, 150 mM NaCl, 0.05% Tween 20, pH: 7.6) and incubated with an HRP-conjugated secondary antibody (Pierce) for 1 h at room temperature. Signals were detected using DAB (diaminobenzidine, Dako Denmark A/S) solution.

### *E. coli* toxicity assays

The wild type and point mutants of *tle1*^KP^ were sub-cloned into the pET-22b vector containing N-terminal PelB signal peptide sequences. The plasmid pET28a was used to express *tle1*^KP^ in the cytoplasm. A single colony harboring the expression plasmid was grown in LB medium at 37°C. After overnight culture to the stationary phase and suspended in LB medium, we diluted strains to an optical density at OD_600_ of 0.2. Then the cells were serially diluted in 10-fold steps and grown in select culture medium with 0.05 mM IPTG and 50 μg/mL ampicillin (or 200 μg/mL kanamycin). The plates were prepared for imaging and plate counts after an additional 20 h incubation at 37°C. The CFU per milliliter of the surviving prey strain was measured by counting single clones (Hu et al., 2014).

### Statistical analysis

Data are shown as mean ± *SD*. The *t*-test and one-way ANOVA were used to compare continuous variables. A *P* < 0.05 was considered statistically significant. Data were analyzed with the R package.

## Results

### Identification of the T6SS gene cluster in *K. pneumoniae* strain HS11286

**Figure 1:**
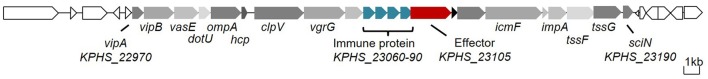
T6SS gene cluster of *K. pneumoniae* HS11286. The core component genes are marked in gray (Supplementary Table 4), the effector gene in red, and the immunity protein genes in blue.

We detected an entire 23-gene T6SS cluster (*KPHS_22970-23190*) on the chromosome of *K. pneumoniae* strain HS11286 (Supplementary Table 4). This T6SS consisted of 12 core components (Figure 1). Interestingly, we found a 4.7 kb insertion region that contained *KPHS_23060-23110*, which is located between the core component genes *icmF* and *vgrG*. There is a sequencing mistake in the insertion region. According to Sanger sequencing of the PCR amplification, a homopolymer error within *KPHS_23100* was made by 454 sequencing. This error resulted in a frame-shift mutation. After correcting the sequencing mistake, the region from *KPHS_23100* to *KPHS_23110* was combined into a new protein-coding gene that we named *KPHS_23105* (Supplementary Figure 4). This gene coded for a putative effector of T6SS that we called Tle1^KP^. This effector contains a conserved alpha/beta hydrolase domain (DUF2235) like the reported T6SS effector Tle1 in *P. aeruginosa* (Hu et al., 2014). A tandem array of four genes (*KPHS_23060-23090*) were predicted to code for immunity proteins Tli1^KP^ that were related to Tle1^KP^. These genes exhibited 94% nucleotide sequence identities to each other. In addition, VRprofile typed 254 putative T6SS gene clusters in the 107 completely sequenced *K. pneumoniae* genomes (including HS11286), of which, 42 of the gene clusters code for homologous effectors of Tle1^KP^ (numbers 1–42 in Supplementary Figure 5).

### T6SS secretion activity is induced by meropenem and ceftazidime

**Figure 2:**
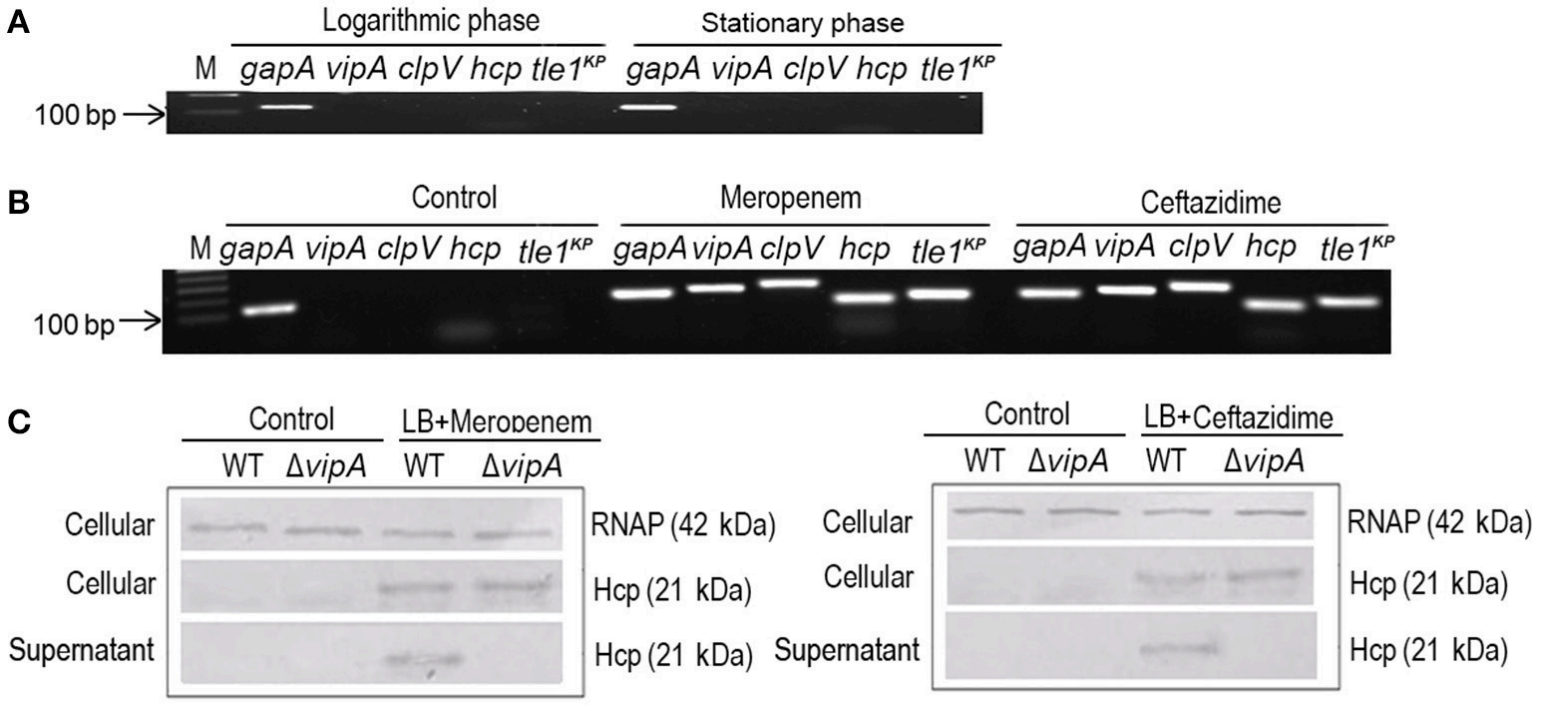
The expression and secretion of T6SS of *K. pneumoniae* HS11286. **(A)** Semi-quantitative PCR was used to detect the expression of T6SS, with *gapA* as the reference gene, and samples were cultured with LB medium. *vipA, clpV, hcp*, and *tle1*^*KP*^ coded for tube sheath, ATPase, hallmark effector, and putative effector, respectively. **(B)** The expression of T6SS of *K. pneumoniae* HS11286 cultured with the addition of the sub-inhibitory concentration of 4 mg/L meropenem or 32 mg/L ceftazidime (Supplementary Figure 8). The gels were cropped from the original images available at the Supplementary Figure 6. **(C)** Immunoblots in the supernatant and cellular fractions of the HS11286 wide-type and Δ*vipA* mutant using specific antibodies against Hcp and RNAP (cellular control).

To characterize the predicted T6SS, we first determined the expression of the T6SS core component and effectors in the logarithmic and stationary phases of *K. pneumoniae* grown in LB medium. The marker genes that we used were *gapA, vipA, clpV, hcp*, and *tle1*^*KP*^, which encode the housekeeping protein GapA, a VipA sheath, an ATPase, a hallmark effector and the Tle1^KP^ effector, respectively. The semi-quantitative PCR assays showed that there was no detectable band of the four marker genes (besides the housekeeping *gapA*) in either the logarithmic or the stationary phase (Figure 2A), suggesting that T6SS was inactive when cultured in LB broth. We also observed that T6SS was inactive in the M9 medium culture conditions (Supplementary Figure 7). Previously, it was reported that T6SS could be induced under certain conditions, such as temperature, pH, and the presence of chitin or with antibiotics (Cerith et al., 2013; Borgeaud et al., 2015). Accordingly, we tested whether the expression of T6SS in strain HS11286 could be induced with a sub-inhibitory concentration of β-lactam antibiotics (meropenem and ceftazidime). As shown in Figure 2B, *vipA, clpV, hcp*, and *tle1*^*KP*^ exhibited strong bright bands in the presence of a sub-inhibitory concentration of meropenem (4 mg/L) or ceftazidime (32 mg/L) compared to control conditions without antibiotics. The determination of sub-inhibitory concentrations of the antibiotics is shown in Supplementary Figure 8.

To further explore whether the T6SS effectors could be secreted in *K. pneumoniae* under sub-inhibitory antibiotic stress, we produced a Δ*vipA* mutant that cannot secrete T6SS effectors due to the lack of the VipA sheath element. Then, we examined Hcp, one of the reported T6SS effectors, in both the cellular and supernatant fractions by immunoblotting. As shown in Figure 2C, Hcp was absent in both the cellular and supernatant fractions of the wild type and Δ*vipA* mutant when cultured with LB broth. In contrast, Hcp was easily detected in the cellular fraction of both the wild type and Δ*vipA* mutant under β-lactam antibiotic stimulation, indicating that the T6SS of HS11286 could be produced upon exposure to a sub-inhibitory concentration of β-lactam antibiotics. We also observed that Hcp was present only in the supernatant of the wild type strain but not in Δ*vipA* mutant (Figure 2C), suggesting that the secretory function of T6SS occurred under antibiotic stimulation. Also, we identified the specificity of antibiotics by using apramycin (Supplementary Figure 9) and got the similar results. It was to say that not only β-lactam antibiotic can induce the secretion of T6SS.

### Tle1^KP^ and Tli1^KP^ are organized into an effector-immunity protein pair

**Figure 3:**
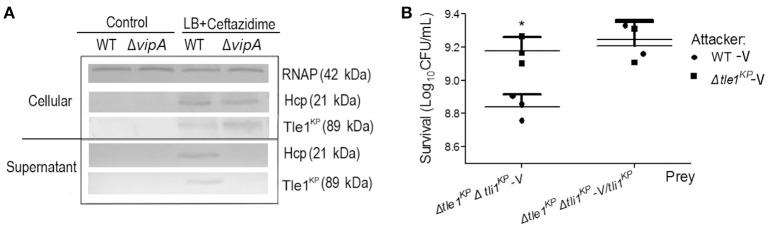
The identification of effector and immune protein pair. **(A)** Immunoblots in the supernatant and cellular fractions of the HS11286 wild-type and Δ*vipA* mutant using specific antibodies against Tle1^KP^, Hcp, and RNAP (cellular control). Samples acquired in liquid culture with 32 mg/L ceftazidime (Supplementary Figure 8). The westernblot membranes were cropped from the original images available at the Supplementary Figure 10. **(B)** Bacterial competition assays. Deletion mutant strain (Δ*tle1*^*KP*^Δ*tli1*^*KP*^*-*V) and complementation of Δ*tle1*^*KP*^Δ*tli1*^*KP*^ mutant with *tli1*^*KP*^ (Δ*tle1*^*KP*^Δ*tli1*^*KP*^-V/*tli1*^*KP*^) as the prey strains following co-culture with the attacking strains (the WT-V and Δ*tle1*^*KP*^ -V mutant, Supplementary Table 1). All strains carried the vector control plasmid pBAD33-Apra. Each group has three replications. ^*^P < 0.05.

Bioinformatics analysis predicted that Tle1^KP^ in HS11286 is an effector of T6SS. To test this, we examined Tle1^KP^ expression in the supernatant of both the wild type and the T6SS-apparatus-deletion mutant (Δ*vipA*) treated with ceftazidime. The results showed that Tle1^KP^ was present in both the cytosol and supernatant fractions of the wild type strain with antibiotic treatment but was absent in the supernatant of the Δ*vipA* mutant strain, indicating that Tle1^KP^ is a T6SS effector (Figure 3A).

The toxicity of Tle1^KP^ was predicted to be neutralized by the upstream cognate immune protein Tli1^KP^. T6SS-dependent killing (dueling) was performed to confirm this prediction, which uses co-incubation of attacker and prey bacterial strains to show whether a predicted immune protein will protect the prey from killing. The attacker and prey strains are co-cultured on agar, and the survival strains are quantified. The double deletion mutant lacking the effector and immune protein pair (strain Δ*tle1*^*KP*^Δ*tli1*^*KP*^-V) was used as the prey that could be susceptible to T6SS. The wild type and Δ*tle1*^*KP*^-V mutant were the attackers. As shown in Figure 3B, the WT-V (Supplementary Table 1) caused a statistically significant impairment/killing of the Δ*tle1*^*KP*^Δ*tli1*^*KP*^*-*V mutant, but the Δ*tle1*^*KP*^-V mutant did not. Additionally, when the prey (Δ*tle1*^*KP*^Δ*tli1*^*KP*^-V) was complemented with *tli1*^*KP*^, the inhibition by the attacker WT-V was same to the attacker Δ*tle1*^*KP*^-V. These results show that the Tli1^KP^ protein counteracts the toxicity of effector Tle1^KP^. Hence, Tle1^KP^ and Tli1^KP^ represent a cognate effector and immunity protein pair.

### HS11286 T6SS-dependent killing within bacterial populations

**Figure 4:**
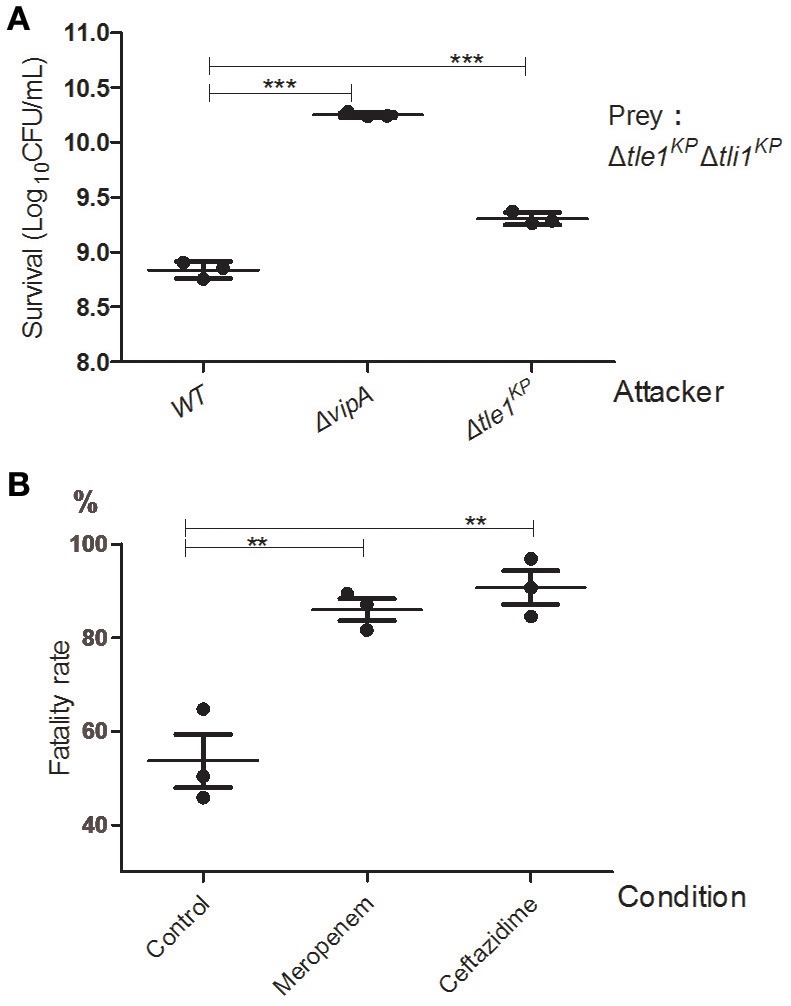
Anti-bacterial activity of the HS11286 T6SS. **(A)** Intraspecies competition assays. Different strains were individually mixed with Δ*tle1*^*KP*^Δ*tli1*^*KP*^ on LB medium and then viability on selective medium was determined. Δ*tle1*^*KP*^Δ*tli1*^*KP*^ was the prey. **(B)** T6SS-dependent killing activity under antibiotic stress. The attacker strains (WT and Δ*vipA*) were individually mixed with the prey strain (Δ*tle1*^*KP*^Δ*tli1*^*KP*^) on different antibiotic media and then titered for viable counts on selective media. The fatality rate was calculated based on viable cell count of the prey with or without competence. *CFU*_competition_/*CFU*_control_. The sub-inhibitory concentrations of meropenem (4 mg/L) and ceftazidime (32 mg/L). Control was added (ddH_2_O). Each group in **(A,B)** has three replications. ^**^P < 0.01; ^***^P < 0.001.

We performed the competition assays to investigate the antibacterial activity of the *K. pneumoniae* HS11286 T6SS. The wild type, the T6SS-apparatus-deletion mutant (Δ*vipA*), and the transferred effector deletion mutant (Δ*tle1*^*KP*^) were employed as the attacker strains. The effector and immune protein gene double deletion mutant strain (Δ*tle1*^*KP*^Δ*tli1*^*KP*^) served as the prey strain. As shown in Figure 4A, the killing activity of the deletion mutant lacking the T6SS apparatus or the effector was lower than that of the wild type (The complementary of Tle1^KP^ in Supplementary Figure 11). The survival of the prey was greater with the Δ*vipA* mutant than with Δ*tle1*^*KP*^, which indicated the T6SS might contain another toxic effector. Also, the fatality rate of the antibiotics-free group (the control) is nearly two-fold less than that of the meropenem or ceftazidime group (Figure 4B). It indicated that the T6SS killing activated by cell-to-cell contact might become stronger under antibiotics stress.

We also confirmed the T6SS-mediated interspecies competition between *K. pneumoniae* and *E. coli*. We utilized *E. coli* DH10B as the prey strain. The HS11286 wild type, the Δ*vipA* mutant, and the Δ*tle1*^*KP*^ mutant were employed as the attacker strains. The attacker strains were individually mixed with the prey and grown on LB agar, and the prey survival was quantified after co-incubation. As expected, the wild type exhibited the strongest killing activity compared to the Δ*vipA* mutant and the Δ*tle1*^*KP*^ mutant (Supplementary Figure 12).

### Overexpression of Tle1^KP^ in the periplasm was lethal in *E. coli*

**Figure 5:**
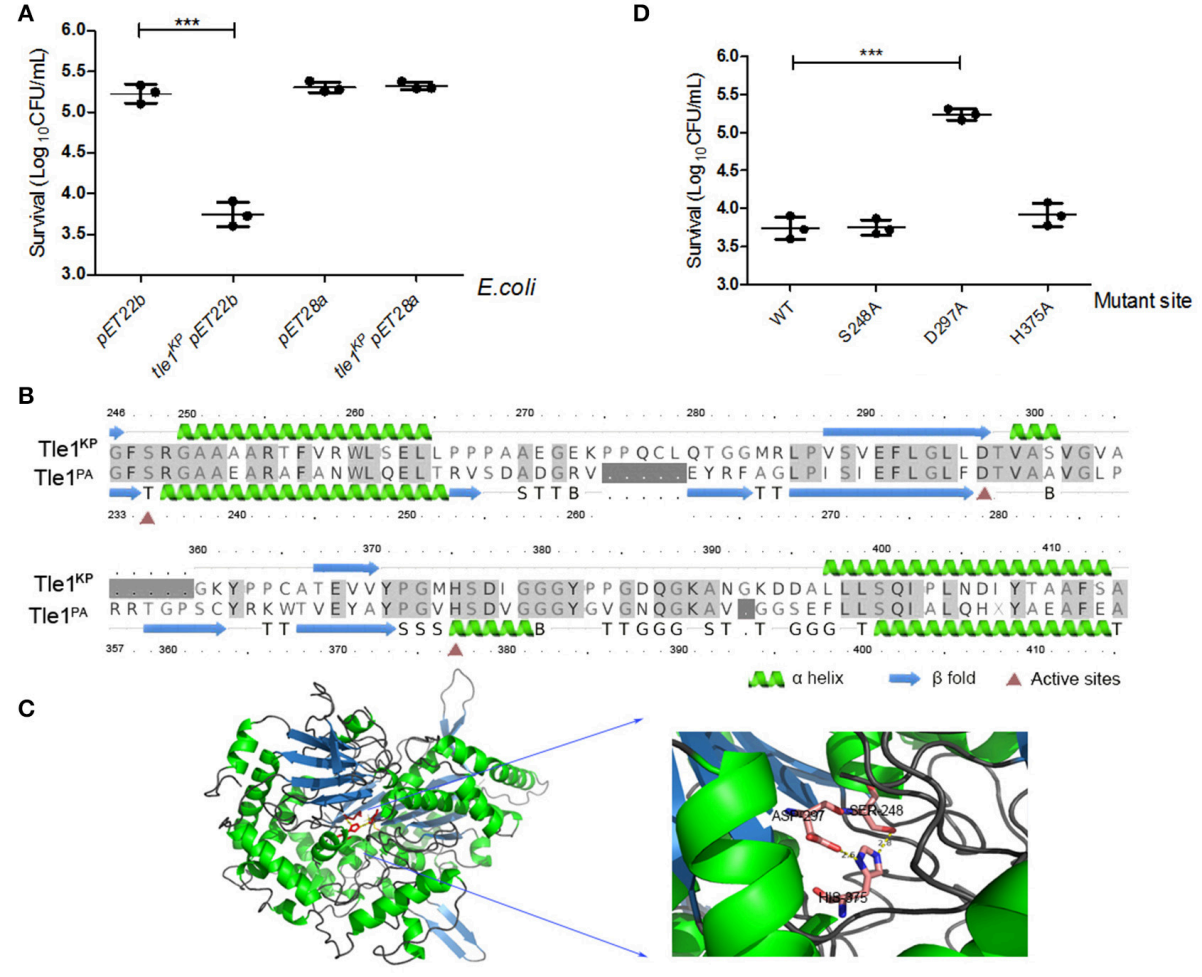
Characterization of the T6SS effector Tle1^KP^. **(A)** The growth of *E. coli* BL21 containing *tle1*^*KP*^ pET22b and *tle1*^*KP*^ pET28a on LB agar induced by IPTG. BL21 with an empty vector was included as a control. pET22b contains a periplasm-targeting signal. Ten-fold serial dilutions of overnight culture were grown on LA media plates with selective culture medium. **(B)** Amino acid sequence alignment for Tle1^KP^ and the Tle1^PA^ of *P. aeruginosa* (Hu et al., 2014). Red triangles highlight the characterized active sites of phospholipase Tle1^PA^. **(C)** The 3D structure and active site of Tle1^KP^ predicted by Phrye2 based on the reported structure of Tle1^PA^ (PDB entry 405p). **(D)** Identification of the active sites of Tle1^KP^. According to the active sites shown in **(C)**, *E. coli* BL21 harboring pET22b expressing wild type and its point mutants in the periplasm were grown in agar plates. The cells were prepared with serial 10-fold dilutions on LA medium and induced by IPTG. Each group in **(A,D)** has three replications. The CFU per milliliter of the surviving prey strain was measured by counting single clones. ^***^P < 0.001.

The Tle1 family effector member from *P. aeruginosa* (Tle1^PA^) can hydrolyze cell membranes (Hu et al., 2014). To confirm the membrane-targeting activity of Tle1^KP^, this effector was produced in *E. coli* strain BL21 and designed to target to the periplasm (Hu et al., 2014). As expected, the presence of Tle1^KP^ in the periplasm inhibited the growth of *E. coli* with significant difference (Figure 5A, *P* = 0.0004), suggesting that Tle1^KP^ is an active membrane-targeting phospholipase effector in *K. pneumoniae*. Meanwhile, no decrease in viability was observed after overexpressing *tle1*^*KP*^ in the cytoplasm (Figure 5A), suggesting that Tle1^KP^ is not toxic in the cytoplasm.

In addition, the structure and active domain of Tle1^PA^ have been reported recently (Hu et al., 2014). The protein sequence alignment between Tle1^KP^ and Tle1^PA^ exhibited 43% identities. According to homology modeling, Tle1^KP^ also contains a catalytic triad (S248-D297-H375), like the one that has been characterized in Tle1^PA^ (Figures 5B,C). Thus, we constructed three *tle1*^*KP*^ mutant strains (S248A, D297A, and H375A) by site-directed mutagenesis for a toxicity assay, as described above. Only the D297A mutant showed an impaired antibacterial effect (Figure 5D, *P* = 0.0003), suggesting that D297 is indispensable in catalytic activity. In Tle1^PA^, site-directed mutations that alter each of these three amino acids lost activity, which implies that Tle1^KP^ and Tle1^PA^ have different active centers that may have different functions.

## Discussion

In this study, we identified and characterized a new T6SS with the antibacterial effector Tle1^KP^ in *K. pneumoniae* HS11286. This T6SS is silent in basic medium without an induction signal. It follows that expression and assembly of this structure would be tightly regulated. In some cases, there is evidence for transcriptional regulation of the T6SS via quorum sensing (Gueguen et al., 2013; Salomon et al., 2013), biofilm formation (Aubert et al., 2008; Hood et al., 2010), iron limitation (Brunet et al., 2011; Chakraborty et al., 2011), and temperature variation (Salomon et al., 2013), which may react to stress responses (Gueguen et al., 2013). In *P. aeruginosa*, kanamycin stimulated T6SS expression but did not affect the cognate effector secretion (Cerith et al., 2013). But in *K. pneumoniae* HS11286, a sub-inhibitory concentration of the β-lactam antibiotics could induce both T6SS expression and secretion of the effectors Hcp and Tle1^KP^.

Furthermore, antibiotics also enhanced the antibacterial activity of the HS11286 T6SS. We propose that antibiotics induce the expression and secretion of T6SS and might make the attacker T6SS^+^ strains more aggressive in the competition for growth. A previous report (Li et al., 2016) has shown a similar phenomenon in *Acidovorax avenae* subsp. avenae (Aaa) strain RS-1. Exposure of RS-1 to ampicillin alters the virulence, colonization capacity, composition of extracellular polymeric substances and secretion of the T6SS effector Hcp. Thus, for CRKP HS11286 in the clinical practice, antibiotics may not inhibit its proliferation but instead may induce the activity of T6SS, making HS11286 more aggressive (Supplementary Figure 13). Under antibiotic stress, CRKP HS1186 thus dominates the growth superiority comparing to the T6SS^−^/multidrug resistant strain.

There are five known Tle family T6SS effectors (Tle1-5; Lu et al., 2014), and the newly identified effector encoded within the T6SS gene cluster of *K. pneumoniae* HS11286 belongs to Tle1. Tle1-Tle4 families contain a conserved G-X-S-X-G motif, and Tle5 features a conserved H-X-K-X-X-X-X-D motif (Durand et al., 2014; Russell et al., 2014). Members of Tle1, Tle2, and Tle5 had been experimentally confirmed to possess phospholipase A2, A1, and D activities, respectively (Durand et al., 2014). After being injected into their periplasmic space by the T6SS, Tle1 of *Burkholderia thailandensis* and Tle2 of *V. cholerae* can hydrolyze the membrane phospholipids of neighboring cells, causing an increase in cellular permeability (Russell et al., 2013; Hu et al., 2014). According to homologous model, Tle1^KP^ of *K. pneumoniae* HS11286 contains a Tle1 family conserved motif G-X-S-X-G and exhibits periplasmic activity. But the active motif of Tle1^KP^ may not be the same as Tle1^PA^, as shown in our point mutations results. And the amino acid sequence identities between reported Tle1^PA^ and Tle1^KP^ were just 43%, also indicating that they might have different active amino acids. Similarly, Tle4 of *P. aeruginosa* possesses an unusual pentapeptide motif T-X-S-X-G (Lu et al., 2014), different from the canonical hydrolases with the (G-X-S-X-G) motif (Durand et al., 2014; Russell et al., 2014).

In conclusion, the anti-bacterial function of the T6SS of *K. pneumoniae* HS11286 was confirmed with the intraspecies and interspecies competition assays. Overexpression of the effector gene *tle1*^*KP*^ in the periplasm caused severe growth retardation of *E. coli*. The results also indicated that the antibiotics could be an important factor for the T6SS secretion and antibacterial activity. To our knowledge, this is the first report about the antibacterial function of T6SS in the *K. pneumoniae*. This information might deepen our understanding of the T6SS-carrying CRKP under antibiotic treatments.

## Author contributions

HO and YY conceived and designed the experiments. LL and XL performed the experiments. LL, XL, MY, JL, HO, and YY analyzed the data. MY, JL, YY, HO, and ZD contributed reagents/materials/analysis tools. LL, YY and HO wrote the paper.

### Conflict of interest statement

The authors declare that the research was conducted in the absence of any commercial or financial relationships that could be construed as a potential conflict of interest.
