# Supplementary material for: Identification and Characterization of an Antibacterial Type VI Secretion System in the Carbapenem-Resistant Strain Klebsiella pneumoniae HS11286
Source: Front Cell Infect Microbiol. 2017 Oct 12;7:442. doi: 10.3389/fcimb.2017.00442 (PMC5649205; doi:10.3389/fcimb.2017.00442)
Supplement: Supplementary file 1 [file Table1.docx]

***Supplementary Materials***

**Identification and characterization of an antibacterial type VI secretion system in the carbapenem-resistant strain *Klebsiella pneumoniae* HS11286**

**Lu Liu^1^, Meiping Ye^2^, Xiaobin Li^1^, Jun Li^1^, Zixin Deng^1^, Yufeng Yao^3^, Hong-Yu Ou^1,^***

*** Correspondence:** Hong-Yu Ou：hyou@sjtu.edu.cn

**Supplementary Tables**

**Supplementary Table 1. Strains used in this study.**

| **Strain** | **Feature** | **Reference** |
| --- | --- | --- |
| *K. pneumoniae* HS11286 | Clinical multidrug-resistant isolate; non-mucoid | (Liu et al., 2012) |
| HS11286-LR | pKOBEG-Apra | This study |
| HS11286-Δ*vipA* | Knock out *KPHS_22970* gene | This study |
| HS11286-Δ*hcp* | Knock out *KPHS_23020* gene | This study |
| HS11286-Δ*tle1^KP^* | Knock out *KPHS_23105* gene | This study |
| HS11286-Δ*tle1^KP^*::hph | Knock out *KPHS_23105* gene, with hygromycin-resistance gene | This study |
| HS11286-Δ*tle1^KP^*Δ*tli1^KP^* | Knock out *KPHS_23060-105* gene | This study |
| HS11286-Δ*tle1^KP^*Δ*tli1^KP^*::hph | Knock out *KPHS_23060-105* gene, with hygromycin-resistance gene | This study |
| HS11286*-*V | Contains pBAD33-Apr | This study |
| HS11286-Δ*tle1^KP^-*V | Knock out *KPHS_23105* gene and contains pBAD33-Apr | This study |
| HS11286-Δ*tle1^KP^*Δ*tli1^KP^-*V | Knock out *KPHS_23060-105* gene and contains pBAD33-Apra | This study |
| HS11286-Δ*tle1^KP^*Δ*tli1^KP^-*V*/tli1^KP^* | Knock out *KPHS_23060-105* gene, and complement KPHS_23060 with *KPHS_23060*/pBAD33-Apra | This study |
| *E. coli* DH10B | clone | Laboratory |
| *E. coli* BL21 | Express protein | Laboratory |

**Supplementary Table 2. Plasmids used in this study.**

| **Plasmid** | **Feature** | **Reference** |
| --- | --- | --- |
| pKOBEG-Apra | Thermo-sensitive replicon (growth at 30°C); λ red genes *gam*, *bet* and *exo* under pBAD promoter (arabinose inducible); Apra^R^ | (Chaveroche. et al., 2000) |
| pJTAG-Hyg | pJTAG-derived; flippase recognition target (FRT)-flanked *hph* cassette; Hyg^R^ | (Bi et al., 2015b) |
| pFLP2-Apra | pFLP2-derived; *sacB flp cI*; Apra^R^ | (Bi et al., 2015b) |
| pET28a | Expression vector with *lacI*, T7 promoter, C-terminal His6 tag, Kan^R^ | Novagen |
| pET22b | Expression vector with *lacI*, T7 promoter, N-terminal PelB signal sequence, C-terminal His6 tag, Amp^R^ | Novagen |
| *tle1^KP^* pET22b | pET22b carrying *KPHS_23105* gene of *K. pneumoniae* HS11286 | This study |
| *tle1^KP^* pET28a | pET28a carrying *KPHS_23105* gene of *K. pneumoniae* HS11286 | This study |
| pBAD33-Apra | Expression vector with pBAD promoter (arabinose inducible); Apra^R^ | (Guzman et al., 1995) |
| *tli1^KP^* pBAD33-Apra | pBAD33 carrying *KPHS_23060* gene of *K. pneumoniae* HS11286 | This study |

**Supplementary Table 3. Oligonucleotides used in this study.**

| **Primer ^a^** | **Sequence (5′ to 3′)** | **Target** |
| --- | --- | --- |
| gapA-RT-F | GCGCTAACTTCGACGCTTAC | HS11286: G2132999-2133123 |
| gapA-RT-R | GGTCATCAGGCCTTCAACGA |  |
| Kp23105-RT-F | AAACGCCCTTCTATCAGCGC | HS11286:G2342539-2342660 |
| Kp23105-RT-R | TGCTATCTGGTTCTGGCGAG | *tle1^KP^* |
| Kp22970-RT-F | CTGCCGCTCAAACTGCTTAC | HS11286: 2326326-2326465 |
| Kp22970-RT-R | GAGAGGTTAACTTCCGGGCT | *gapA* |
| Kp23020-RT-F | GTGAATCCCCTGATGCACGA | HS11286: 2332400-2332489 |
| Kp23020-RT-R | GGTGATCTTCTCGTAGCGCA | *hcp* |
| Kp23030-RT-F | TACGCCGTCTTAATCCCTGC | HS11286: 2332839-2333013 |
| Kp23030-RT-R | CACAGCGCATCCATATCCCA | *clpV* |
| GmF | CGAATTAGCTTCAAAAGCGCTCTGA | pJTAG-Hyg: *hph* cassette |
| Gm-R2 | AATTGGGGATCTTGAAGTTCCT |  |
| Kp22970-soe-s-F | TTAGATATAGCCTGGCGACTTGCA | Reverse-complement to GmF; HS11286: G2325626-2326244 |
| Kp22970-soe-s-R | TCAGAGCGCTTTTGAAGCTAATTCG ATCAGCCATAGCAGTTCCTTTC |  |
| Kp22970-soe-x-F | AGGAACTTCAAGATCCCCAATTCCGAAATAAGTGCGGGGCA | Reverse-complement to Gm-R2; HS11286: G2326719-2327225 |
| Kp22970-soe-x-R | CTCAAAGTCCTGGCGCAGA |  |
| Kp23060-105-S-F | TTATTGTCGATGATGCTCCGCT | Reverse-complement to GmF; HS11286: G2338008-2338621 |
| Kp23060-105-S-R | TCAGAGCGCTTTTGAAGCTAATTCG TCATTCTGAAGGTCTCTCACTG |  |
| Kp23060-105-X-F | AGGAACTTCAAGATCCCCAATTCGA CAACGGTCTGAACGCG | Reverse-complement to Gm-R2; HS11286: G2343428-2343990 |
| Kp23060-105-X-R | CATTGCTGTTGTTCGTATGCCA |  |
| Kp23020-S-F | TGTCAGGGTGCTGCGTGAA | Reverse-complement to GmF; HS11286: G2331456 -2332030 |
| Kp23020-S-R | TCAGAGCGCTTTTGAAGCTAATTCG CATCATCCTGGGATGACGC |  |
| Kp23020-X-F | AGGAACTTCAAGATCCCCAATTCGTCATATT TCGCGCCGCT | Reverse-complement to Gm-R2; HS11286: G 2332558 -2333151 |
| Kp23020-X-R | ACACTGCGGATCTGCGCTT |  |
| Kp23105-p-F | GGGGAATTCGATGTCCGAAATAACTGAAACCCATGCCG | HS11286: G2340998-2343416 |
| Kp23105-p-R | GGGCTCGAGCAGCCAGCTGGCCGG CAGCG |  |
| Kp23060-P-F | GGGGAATTCGATGAAATCTGCATTAATCAGCCCGC | HS11286: G2342120-2343418 |
| Kp23060-P-R | GGCTCGAGCTACTTCGGACAGACGGCCG |  |
| Kp23105-297A-F | AGTTTCTCGGGCTGCTGGCTACGGTGGCCTCCG | HS11286: G2340998-2343416 |
| Kp23105-297A-R | CGGAGGCCACCGTAGCCAGCAGCCCGAGAAACT |  |
| Kp23105-197A-F | AGAAATGAGCACCAGCGCGAACCGGCTG TGGTTTGGC | HS11286: G2340998-2343416 |
| Kp23105-197A-R | GCCAAACCACAGCCGGTTcgcGCTGGTGCTCATTTCT |  |
| Kp23105-374A-F | GTGGTTTACCCGGGGATGGCCTCGGATATTGGTGGA | HS11286: G2340998-2343416 |
| Kp23105-374A-R | TCCACCAATATCCGAGGCCATCCCCGGGTAAACCAC |  |
| Kp23105-248A-F | TGTATGTCTATGGTTTTGCCCGCGGCGCGGCAG | HS11286: G2340998-2343416 |
| Kp23105-248A-R | CTGCCGCGCCGCGGGCAAAACCATAGACATACA |  |

^a^ F indicates the forward primer, and R indicates the reverse primer.

**Supplementary Table 4. The core components encoded by the T6SS gene cluster of *K. pneumoniae* HS11286 identified by VRprofile.**

| **ORF** | **T6SS core component** | **COG** | **Note** |
| --- | --- | --- | --- |
| *[KPHS_22970](http://202.120.12.133/SecReT6/component.php?pid=378978456)* | TssB (VipA) | COG3516 |  |
| *[KPHS_22980](http://202.120.12.133/SecReT6/component.php?pid=378978457)* | TssC (VipB) | COG3517 |  |
| *[KPHS_22990](http://202.120.12.133/SecReT6/component.php?pid=378978458)* | TssK (VasE) | COG3522 |  |
| *[KPHS_23000](http://202.120.12.133/SecReT6/component.php?pid=378978459)* | TssL (DotU) | COG3455 |  |
| *KPHS_23010* | - | - | OmpA/MotB domain-containing protein |
| *[KPHS_23020](http://202.120.12.133/SecReT6/component.php?pid=378978459)* | TssD (Hcp) | COG3157 |  |
| *[KPHS_23030](http://202.120.12.133/SecReT6/component.php?pid=378978462)* | TssH (ClpV) | COG0542 |  |
| *[KPHS_23040](http://202.120.12.133/SecReT6/component.php?pid=378978463)* | TssI (VgrG) | COG350z1 |  |
| *KPHS_23050* | - | - | hypothetical protein |
| *KPHS_23060* | - | - | immunity protein Tli1^KP^ |
| *KPHS_23070* | - | - | immunity protein Tli1^KP^ |
| *KPHS_23080* | - | - | immunity protein Tli1^KP^ |
| *KPHS_23090* | - | - | immunity protein Tli1^KP^ |
| *KPHS_23105* | - | - | The phospholipase Tle1^KP^ (Supplementary Figure 3) |
| *KPHS_23120* | - | - | PaaR repeat-containing protein |
| *KPHS_23130* | - | - | hypothetical protein |
| *[KPHS_23140](http://202.120.12.133/SecReT6/component.php?pid=378978473)* | TssM (IcmF) | COG3523 |  |
| *[KPHS_23150](http://202.120.12.133/SecReT6/component.php?pid=378978474)* | TssA (ImpA) | COG3515 |  |
| *[KPHS_23170](http://202.120.12.133/SecReT6/component.php?pid=378978476)* | TssF | COG3519 |  |
| *[KPHS_23180](http://202.120.12.133/SecReT6/component.php?pid=378978477)* | TssG | COG3520 |  |
| *[KPHS_23190](http://202.120.12.133/SecReT6/component.php?pid=378978478)* | TssJ (sciN) | COG3521 |  |

**Supplementary Figures**

**
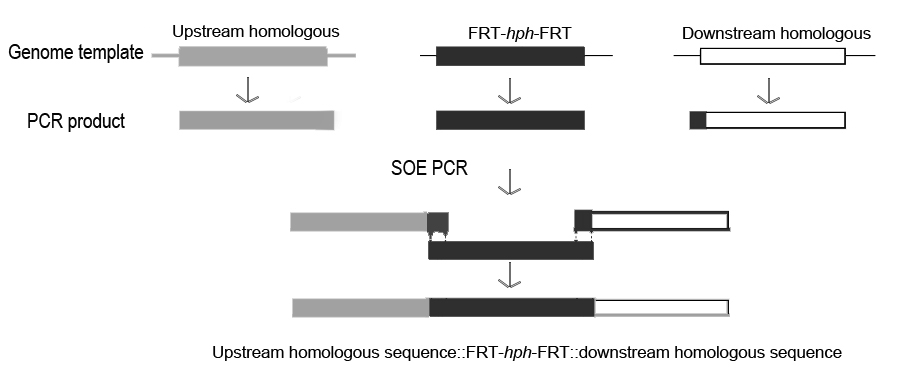
**

**Supplementary Figure 1. Overlap extension PCR (SOE PCR).** Overlap extension PCR to build an FRT site-flanking *hph* cassette. The upstream or downstream homologous sequences are ~ 500 bp in size. The final product is the DNA fragment containing the upstream homologous sequence::FRT-*hph*-FRT::downstream homologous sequence.

**
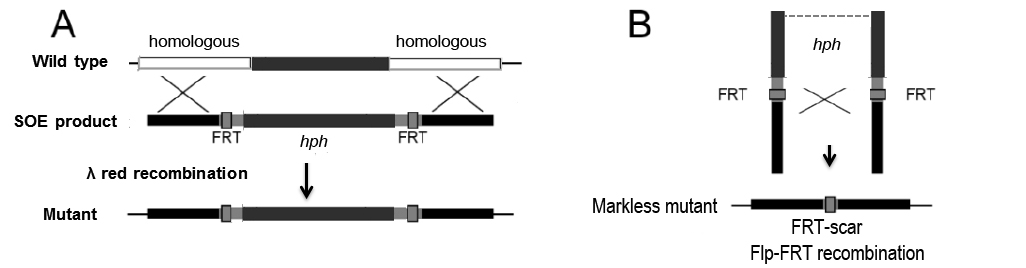
**

**Supplementary Figure 2.** (A) λ red recombination by using the plasmid pKOBEG (Supplementary Table 2). (B) Flp-FRT recombination by using the plasmid pFLP2-Apr (Supplementary Table 2).


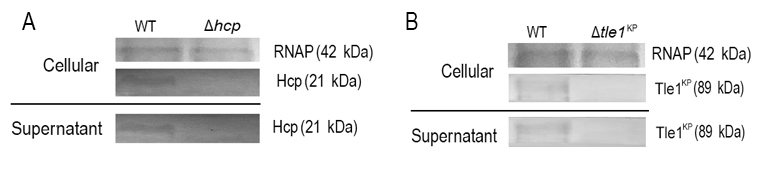


**Supplementary Figure 3. The specificity of the antibody.** Immunoblots in the supernatant and cellular fractions of the HS11286 wide-type, the Δ*hcp* mutant and the Δ*tle1^KP^* mutant to check the specificity of the antibody. RNAP was the cellular control.


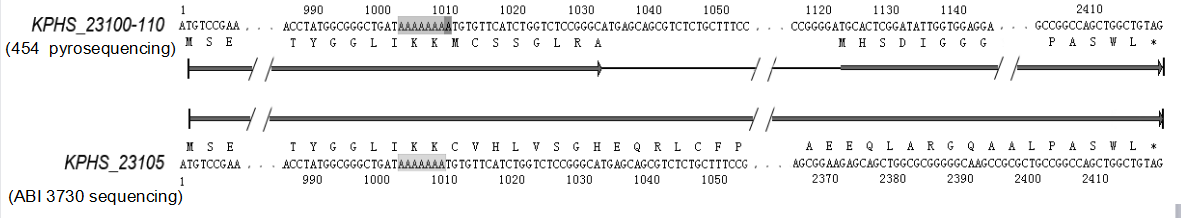


**Supplementary Figure 4. Examination of the 454 sequencing homopolymer-length error within the *KPHS_23100* gene of *K. pneumoniae* HS11286.** The change from 8-mer “A” to 7-mer “A” resulted in the combination of the region from *KPHS_23100* to *KPHS_23110* into a protein-coding region that we named *KPHS_23105*.

**
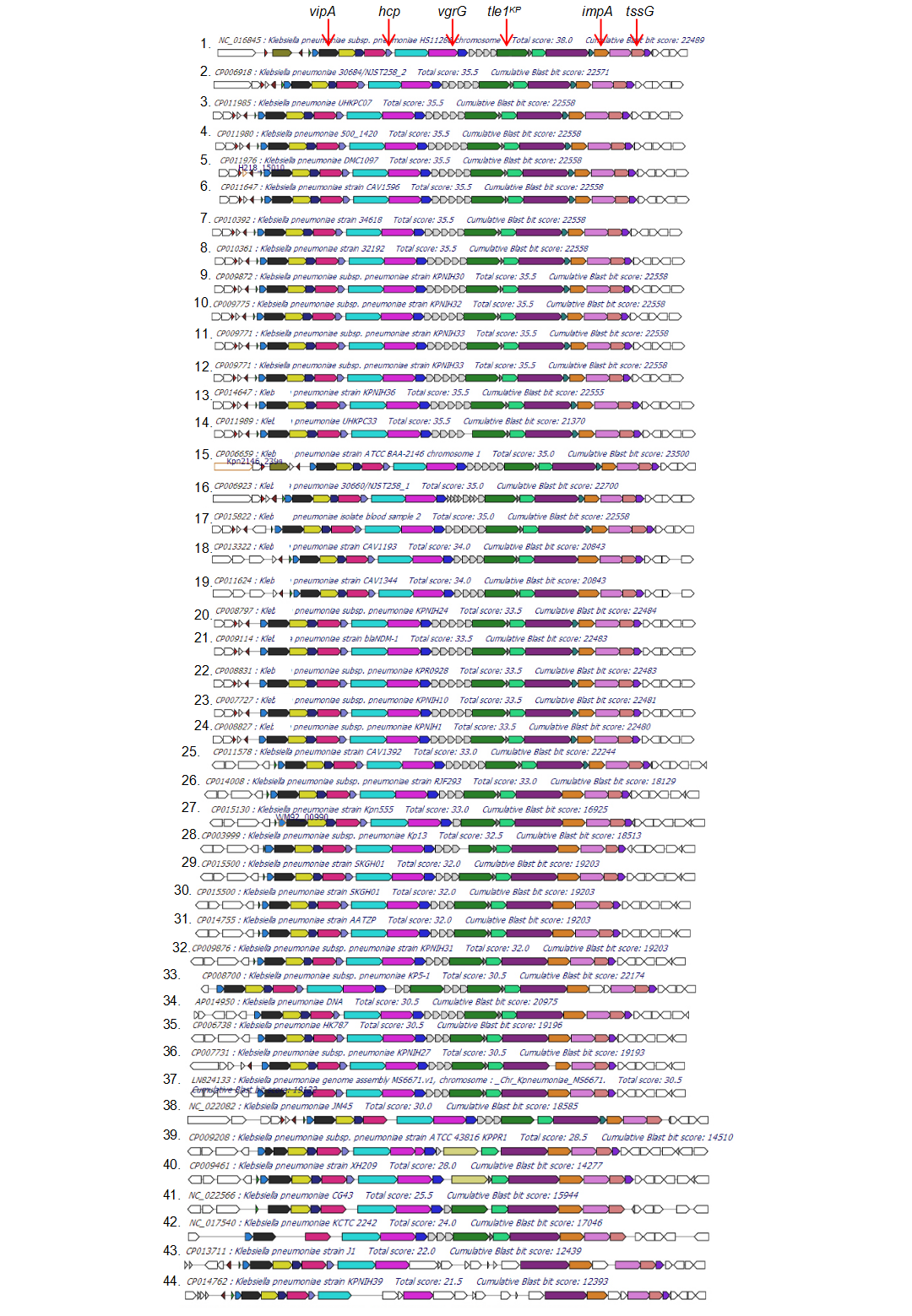
**

**Supplementary Figure 5. Comparison of the T6SS gene cluster of *K. pneumoniae* HS11286 with the other 44 *K. pneumoniae* genomes.** VRprofile typed 254 putative T6SS gene clusters in the 107 completely sequenced *K. pneumoniae* genomes (including HS11286), and 42 T6SS gene clusters coded for homologues of the effector Tle1^KP^ (No. 1 - 42). The CGCfinder tool of VRprofile (Bi et al., 2015b) was used to perform the MultiGeneBlast-facilitated T6SS gene cluster alignments based on protein sequence similarity and gene order. The score was calculated based on the number of BLASTp hits matching the default thresholds set and the number of adjacent gene pairs with conserved synteny. The homologous components are marked with the same color.


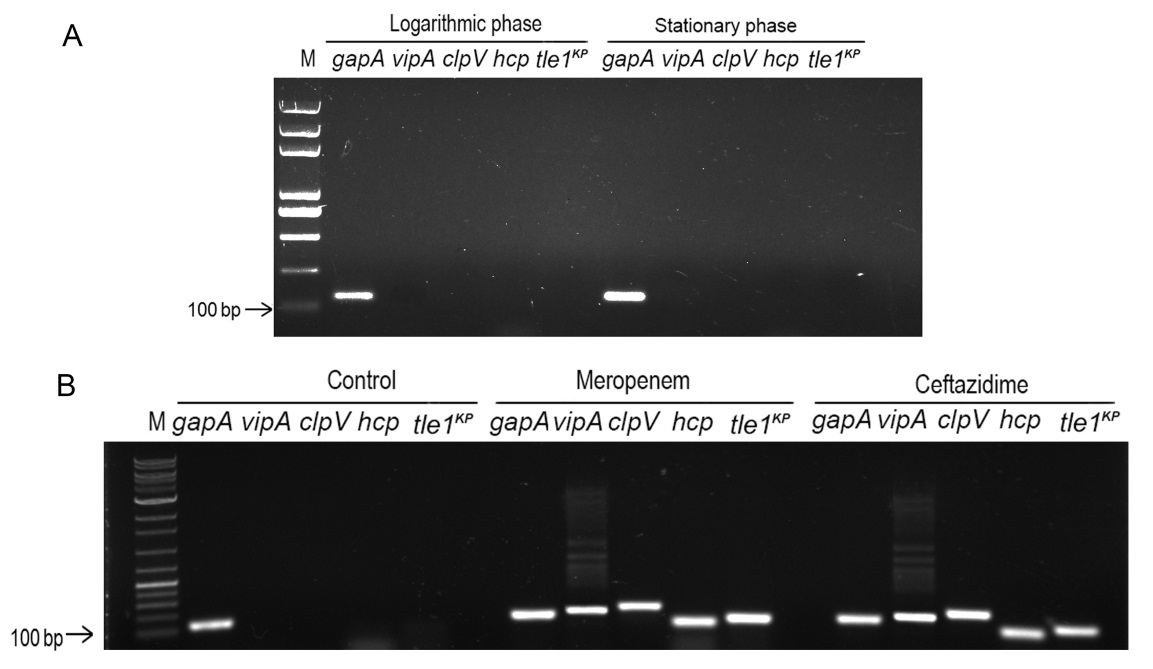


**Supplementary Figure 6. Original images of cropped gels used in Figure 2A-B. (A)** Semi-quantitative PCR was used to detect the expression of T6SS, with *gapA* as the reference gene, and samples were cultured with LB medium. *vipA, clpV, hcp,* and *tle^KP^* are tube sheath, ATPase, hallmark effector and putative effector, respectively. **(B)** The expression of T6SS of *K. pneumoniae* HS11286 cultured with the addition of the sub-inhibitory concentration of 4 mg/L meropenem or 32 mg/L ceftazidime (Supplementary Figure8).


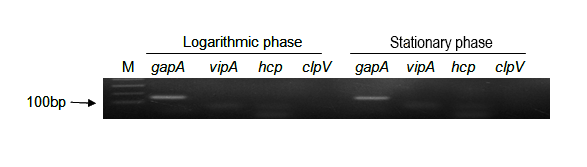


**Supplementary Figure7. Expression of HS11286 T6SS core component genes in M9 medium.** No T6SS gene was expressed in M9 in either the logarithmic or stationary phase (with *gapA* as the control).


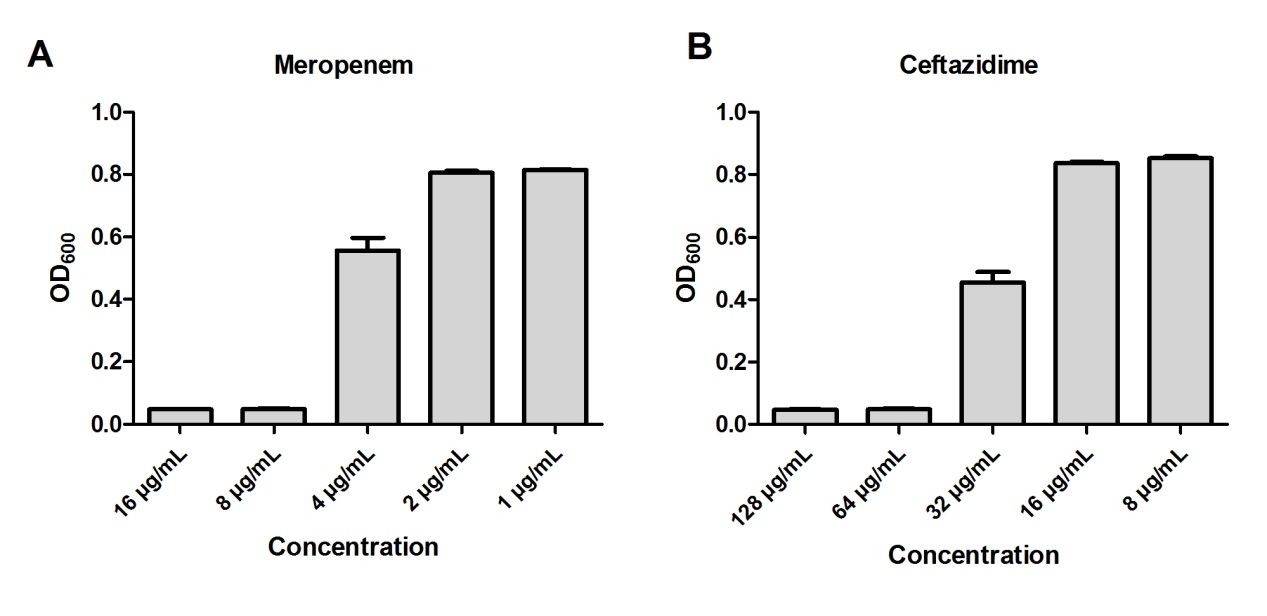


**Supplementary Figure 8. Determination of sub-inhibitory concentrations of the antibiotics.** Based on the minimum inhibitory concentration (MIC) of HS11286 (Bi et al., 2015a), we chose concentrations near the MIC to detect the sub-inhibitory concentration of meropenem **(A)** and ceftazidime **(B)** in a 96-well plate. OD_600_ was measured after strain were grown for 4 h. The sub-inhibitory concentration of antibiotics does not completely inhibit growth of the strains.


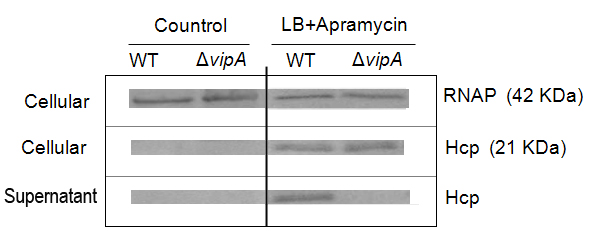


**Supplementary Figure 9.** Immunoblots in the supernatant and cellular fractions of the HS11286 wide-type and Δ*vipA* mutant. The specific antibodies were used against Hcp and RNAP (cellular control). Apramycin: 2 μg/mL.


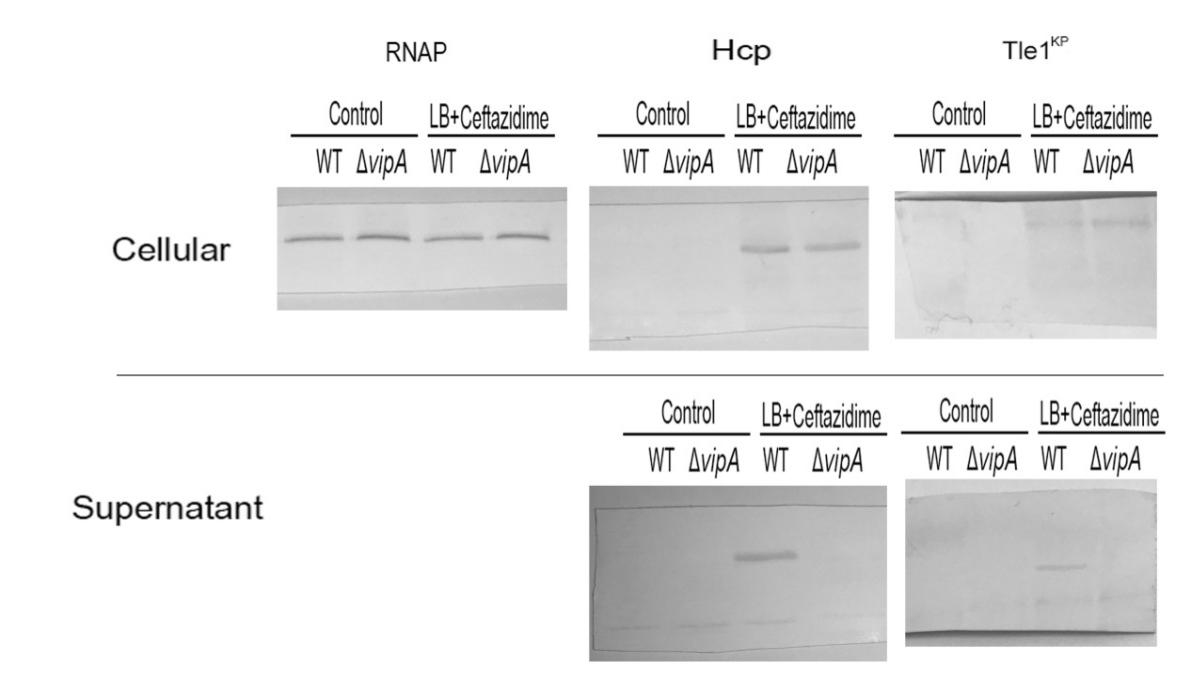


**Supplementary Figure 10. Original images of cropped gels used in Figure 3A.** Immunoblots in the supernatant and cellular fractions of the HS11286 wide-type and Δ*vipA* mutant using specific antibodies against Tle1^KP^, Hcp and RNAP (cellular control). Samples acquired in liquid culture with 32 mg/L ceftazidime (Supplementary Figure 8).


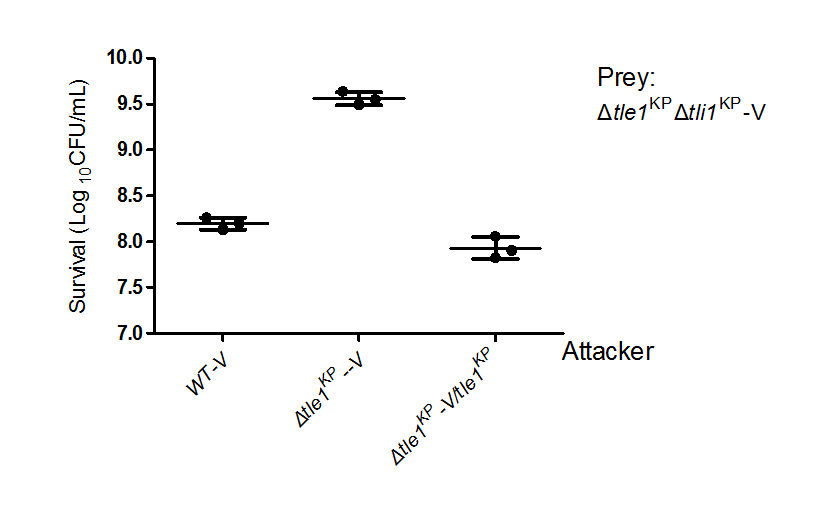


**Supplementary Figure 11. Δ*tle1^KP^* mutant complemented with *tle1*^KP^ in competition assays.** Different strains were individually mixed with Δ*tle1^KP^*Δ*tli1^KP^* on the LB medium and then the viability on selective medium was determined. Δ*tle1^KP^*Δ*tli1^KP^* was the prey. All strains carried the vector control plasmid pBAD33-Apra.


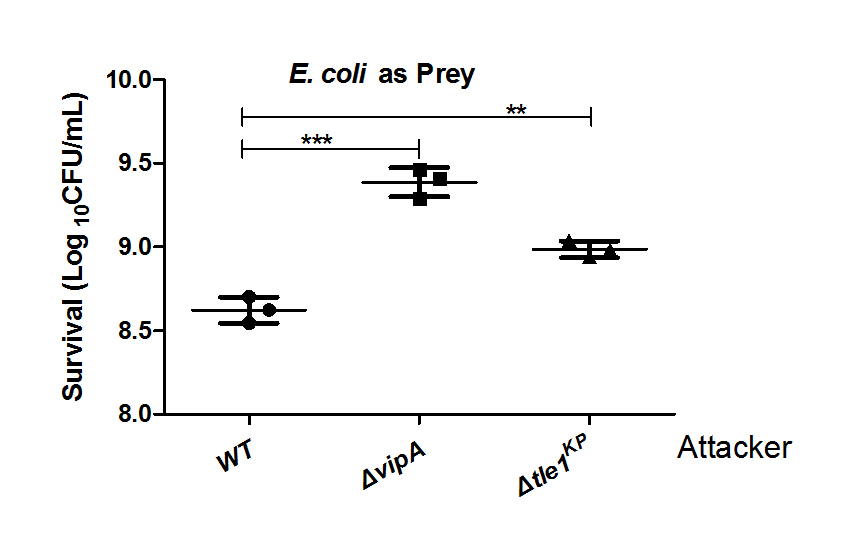


**Supplementary Figure 12. Interspecies competition assay of growth on LB medium.** The *K. pneumoniae* HS11286 wild type, the T6SS-apparatus-deletion mutant (Δ*vipA*) and the transferred effector deletion mutant (Δ*tle1^KP^*) were employed as the attacker strains. *E. coli* DH10B was the prey strain. The selected plates for counting CFU of prey *E. coli* DH10B contained streptomycin. **: *P<*0.01; ***: *P<*0.001.


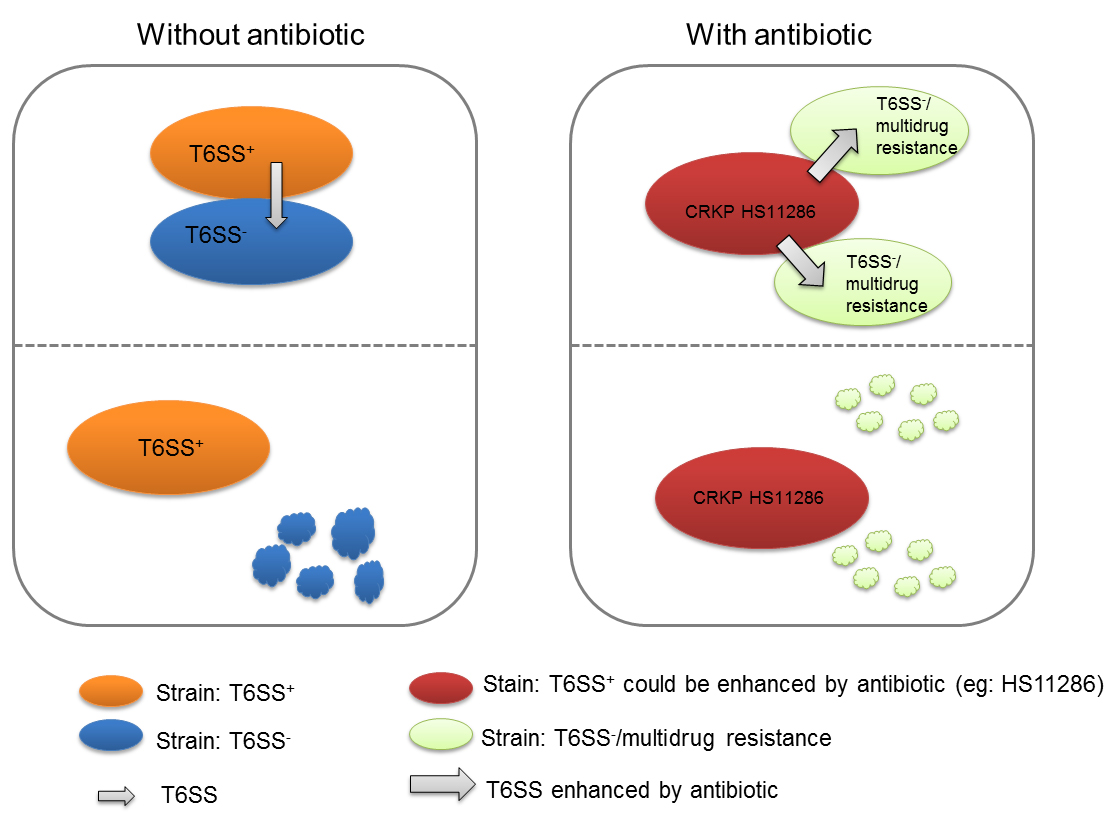


**Supplementary Figure 13. Growth advantages of T6SS^+^strains with or without antibiotic stress.** (A) Without antibiotic condition. The T6SS^+^ strains dominate in this environment while the T6SS^-^ strain might be killed by T6SS. (B) With antibiotic situation. The HS11286 strain with the enhanced T6SS might have more growth advantages. More T6SS^-^ strains were killed, including the multidrug resistant strains without T6SS.
